# Retirement Factors Driving South Korea’s Highest Older Adult Poverty Rate Among OECD Nations: A Decomposition Analysis

**DOI:** 10.1093/geroni/igaf122.2760

**Published:** 2025-12-31

**Authors:** Seoyeon Ahn, Eunsun Kwon, Ji Young Kang, Sojung Park

**Affiliations:** Duke-NUS Medical School/National University of Singapore, Singapore, Singapore; Fairleigh Dickinson University, Florham Park, New Jersey, United States; Chungnam National University, Daejeon, Republic of Korea; Washington University in St. Louis, St. Louis, Missouri, United States

## Abstract

This study investigates why South Korea has the highest older adult poverty rate (40%) among OECD countries by decomposing poverty gaps between Korea and other nations. Using data from the Luxembourg Income Study, we apply the Oaxaca-Blinder decomposition method to compare Korea with eight OECD countries (Norway, Germany, Greece, the United States, the United Kingdom, Canada, Australia, and Japan). Our findings reveal that 84–100% of the poverty rate differences are explained by structural factors. If Korea had Germany’s socioeconomic structure, its poverty rate would drop from 51.9% to 5.8%. Korea’s high older adult employment rate helps reduce poverty by 3.9–7.9 percentage points compared to countries like Japan and Australia. At the same time, Korea’s extensive private transfer income lowers poverty by 2.9–4.9 percentage points. However, the most significant factor driving Korea’s high poverty rate is its inadequate public pension system. If Korea’s public pension benefits aligned with those of developed nations, poverty would decline by 24.1–50.2 percentage points. Despite mitigating effects from labor market participation and private transfers, insufficient public pension income remains the dominant cause of older adult poverty in Korea. These findings highlight the urgent challenge of ensuring income security amid rapid population aging and low fertility rates.

